# Structural Properties and Thermoelectric Performance of the Double-Filled Skutterudite (Sm,Gd)*_y_*(Fe*_x_*Ni_1-*x*_)_4_Sb_12_

**DOI:** 10.3390/ma12152451

**Published:** 2019-08-01

**Authors:** Cristina Artini, Riccardo Carlini, Roberto Spotorno, Fainan Failamani, Takao Mori, Paolo Mele

**Affiliations:** 1 Department of Chemistry and Industrial Chemistry, University of Genova, Via Dodecaneso 31, Genova 16146, Italy; 2 Institute of Condensed Matter Chemistry and Technologies for Energy, National Research Council, CNR-ICMATE, Via De Marini 6, Genova 16146, Italy; 3 National Institute for Materials Science (NIMS), International Center for Materials Nanoarchitectonics (MANA) and Center for Functional Sensor & Actuator (CFSN), Namiki 1-1, Tsukuba 305-0044, Japan; 4 University of Tsukuba, Graduate School of Pure and Applied Sciences, 1-1-1 Tennoudai, Tsukuba 305-8671, Japan; 5 Shibaura Institute of Technology, Omiya Campus, 307 Fukasaku, Minuma-ku, Saitama City, Saitama 337-8570, Japan

**Keywords:** thermoelectricity, skutterudites, crystal structure, powder x-ray diffraction, thermal conductivity

## Abstract

The structural and thermoelectric properties of the filled skutterudite (Sm,Gd)*_y_*(Fe*_x_*Ni_1-*x*_)_4_Sb_12_ were investigated and critically compared to the ones in the Sm-containing system with the aim of unravelling the effect of double filling on filling fraction and thermal conductivity. Several samples (*x* = 0.50–0.90 and *y* = 0.15–0.48) were prepared by melting-sintering, and two of them were densified by spark plasma sintering in order to study their thermoelectric features. The crystallographic study enables the recognition of the role of the filler size in ruling the filling fraction and the compositional location of the *p*/*n* crossover: It has been found that the former lowers and the latter moves toward lower *x* values with the reduction of the filler ionic size, as a consequence of the progressively weaker interaction of the filler with the Sb_12_ cavity. The analysis of thermoelectric properties indicates that, despite the Sm^3+^/Gd^3+^ small mass difference, the contemporary presence of these ions in the 2*a* site significantly affects the thermal conductivity of both *p*- and *n*-compositions. This occurs by reducing its value with respect to the Sm-filled compound at each temperature considered, and making the overall thermoelectric performance of the system comparable to several multi-filled (Fe, Ni)-based skutterudites described in the literature.

## 1. Introduction

In the field of modern solid state chemistry and physics, thermoelectricity occupies a central role due to its relevance in the framework of energy conversion, in particular, refrigeration and electric power generation. Moreover, energy harvesting to power myriad sensors and devices for the Internet of Things (IoT) society is also a promising application [1,2]. The development of thermoelectric technology exploitable for large-scale employments implies in the first instance the search for materials fulfilling several requirements, such as high electrical conductivity and low thermal conductivity. The dimensionless thermoelectric figure of merit *ZT* can be in fact expressed as follows:

(1) $$ZT=\frac{\sigma S^{2}}{\lambda_{e}+\lambda_{ph}+\lambda_{bp}}\;\;$$

where *T* is the absolute temperature, σ the electrical conductivity, S the Seebeck coefficient, $\lambda_{el}$, $\lambda_{ph}$ and $\lambda_{bp}$ the electron, phonon and bipolar [3,4] contributions to thermal conductivity, with $\lambda_{ph}$ being the prevailing one in semiconductors, and $\lambda_{bp}$ appearing only above a certain temperature threshold which depends on the system considered. Electrical conductivity and electron thermal conductivity are correlated through the Wiedemann-Franz law, and for this reason they cannot be separately manipulated. The need for decoupling thermal and electrical conductivity suggests that a two-fold strategy is pursued, consisting of mainly acting on the reduction of $\lambda_{ph}$ and the increase of σ. This goal can be achieved by addressing the search for valid thermoelectric materials for semiconductors. In semiconductors, in fact, at variance with metals, σ increases with increasing temperature, and the quantity $\sigma S^{2}$ (also called the power factor) presents a maximum within their carrier density range. Moreover, $\lambda_{ph}$ prevails over $\lambda_{e}$, making it possible to separately act on the two terms.

While the improvement of the power factor needs efforts in band structure engineering in order to optimize the carrier concentration [5,6], the manipulation of $\lambda_{ph}$ has been of the focus researchers for a long time, since this parameter is considered the easiest one to be addressed by a phenomenological approach.$\;\lambda_{ph}$ can be described by the following Equation:

(2) $$\lambda_{ph}=\frac{1}{3}C_{v}l$$

where $C_{v}$ is the specific heat at a constant volume, l is the mean free path of phonons, and v is the sound velocity. The reduction of l, leading to the reduction of $\lambda_{ph}$, can be achieved through the introduction of scattering centers into the lattice which are able to interfere with phonon transmission, such as the point and volume defects (by alloying) [7,8], grain boundaries (by nanostructuring) [9], nanoinclusions [10,11] secondary phases [12,13], vacancies causing mass fluctuations [14], nano- and micro-sized pores [15,16], or loosely bound atoms.

The last mentioned issue, mainly ruled by the phonon glass electron crystal (PGEC) theory [17], opened the way to a widespread investigation of intermetallic compounds characterized by a high electrical conductivity coupled to a crystal structure provided with large voids, and able to host foreign atoms of proper size. According to the basic concept of the aforementioned theory, in fact, such materials are expected to exhibit a dramatic decrease of phonon thermal conductivity due to the rattling movement of host ions within the cage, without affecting the mobility of charge carriers. These features are actually displayed by members of several classes of intermetallic compounds, such as skutterudites [18], clathrates [19,20], and half-Heusler phases [21]. Skutterudites MX_3_ [18,22] (where M is a transition metal, such as Co, Fe, Rh or Ir and X a pnicogen atom), in particular, exhibit a body-centered cubic cell (Pearson symbol *cI*32, $Im\bar{3}$ space group, isotypic crystal: CoAs_3_) presenting two distinct atomic sites: the 8*c* (¼, ¼, ¼) and the 24*g* (0, *y*, *z*), occupied by M and X, respectively. Consequently, M forms with X a strongly tilted corner-sharing octahedra, and a X_12_ icosahedral cage with its center in the 2*a* site located in (0, 0, 0) appears. The occurrence of the cited cavity is of primary importance for the thermoelectric performance of the material [23]. The skutterudite itself, in fact, is characterized by an excessively high value of thermal conductivity to be reasonably employed for thermoelectric applications [24]. On the contrary, when an atom of proper size is placed into the void, $\lambda_{ph}$—and hence thermal conductivity—is strongly lowered due to the vibrational modes of the rattling guest atom, which disturb the propagation of heat-carrying phonons, as experimentally observed [25]. Clearly, the size of the guest atom must fit the cavity, namely it has to be large enough to be retained within the void, but at the same time small enough to properly vibrate around its equilibrium position. Ideally, in order to maximize the effect of the $\lambda_{ph}$ reduction, even constraints on the atomic mass of the filler should be obeyed. Generally speaking, a large atomic mass is preferable in order to favor low frequency vibrational modes. Considering both the size and the mass requirements, the most suitable atoms are lanthanides and alkaline-earth elements [18,22,26].

It is noteworthy that, in addition to the $\lambda_{ph}$ reduction, the introduction of the filler ion also exerts a significant effect on the electronic properties of the material. While in fact the skutterudite CoSb_3_ is a compensated semiconductor, the substitution of Co by the mixture Fe/Ni causes an electronic imbalance, which is only partly restored by the insertion into the structure of the filler ion. It is generally accepted that a larger or smaller filler amount than the one corresponding to the structural and electronic stability of the resulting compound can be hardly forced into the cavity [18]. The dependence of the Yb solubility in CoSb_3_ on the thermal treatment temperature, for instance, has been recently discussed [27]. However, a large number of studies demonstrated that the filling fraction of the voids depends in the first instance on the oxidation state of the filler, and secondly, on its ionic size, and therefore, it cannot be ad libitum varied. In the RE*_y_*(Fe*_x_*Ni_1-*x*_)_4_Sb_12_ systems (RE ≡ rare earth ion), for example, the filling fraction by trivalent lanthanide ions follows a linear trend as a function of *x*, with different fillers presenting at least roughly comparable amounts at a given Fe/Ni content, and small differences attributable to their different ionic sizes [28,29,30,31,32,33,34,35].

An even more effective approach aimed at the depression of $\lambda_{ph}$ consists of the introduction of a structural disorder through the substitution of Co by Fe/Co [36,37,38,39], Co/Ni [40,41] Fe/Ni [29,34,38,42,43] or Fe/Co/Ni [30,31], the substitution of Sb by Sb/Sn or Sb/Ge [36], or multiple filling of the cavity by DD (didymium) [36,38,41], Mm (mischmetal) [31,41], Ce/Yb [30], Ce/Nd [44], Ce/Yb/In [45] or by a mixture of alkaline-earths [46] with the possible addition of lanthanide elements [41,42] and elements of the IIIB group [47]. Multiple filling, in particular, was thoroughly studied [48], since it ensures a broadening of the range of phonon frequencies which can be perturbed by the rattling movement of doping ions. For this reason, when designing a multiple doping, a significant difference in the mass of the different fillers is generally recommended.

In recent years, the synthetic procedure [49], the room [34] and high temperature [35] structural properties, as well as the thermoelectric [50,51], thermal [52] and mechanical [53] features, and the corrosion behavior [54] of the Sm*_y_*(Fe*_x_*Ni_1-*x*_)_4_Sb_12_ system, were extensively studied by the present research group. In this work, the addition of a second filler element has been attempted in order to lower thermal conductivity and hence to improve ZT. Gd was chosen taking into account its larger mass and slightly smaller size with respect to Sm. The structural properties of the obtained double-filled skutterudite are analyzed relying on powder x-ray diffraction data mainly in comparison to the only Sm-doped compound. The thermoelectric performance is discussed in the light of literature data collected on multiple-doped (Fe,Ni)-based skutterudites.

## 2. Materials and Methods

### 2.1. Synthesis

Five compositions belonging to the (Sm,Gd)*_y_*(Fe*_x_*Ni_1-*x*_)_4_Sb_12_ system, with nominal *x* = 0.90, 0.80, 0.70, 0.63 and 0.50, were synthesized by the direct reaction of pure elements Fe (Alfa-Aesar, 99.99 wt %), Ni, Sm, Gd (NewMet, 99.9 wt %) and Sb (Mateck, 99.999 wt %). The compositions were chosen in order to have *p*- (*x* = 0.90, 0.80, 0.70) and *n*-type (*x* = 0.50) samples, as well as a composition located in the *p*/*n* crossover region (*x* = 0.63). As aforementioned, the filler content is strictly related to the electronic count of the compound, and hence to the Fe/Ni ratio. For this reason, the total rare earth amount to be used (*y*) was determined relying on the results of a previous study performed on the Sm*_y_*(Fe*_x_*Ni_1-*x*_)_4_Sb_12_ system [34]. Moreover, considering the larger size of Sm^3+^ with respect to Gd^3+^, which is expected to allow a better accommodation of the former within the cavity, the 2:1 ratio for the Sm:Gd amount was chosen. A slight Sb excess with respect to the stoichiometric amount was used due to its non-negligible vapor pressure (10^−3^ mm Hg at 873 K [55]). The elements mixture was placed into an Ar-filled quartz ampoule, which was then sealed under an Ar flow, thermally treated at 1223 K for 3 h, and subsequently rapidly cooled in an iced water bath. The obtained samples were then annealed at 873 K for 4 days.

Afterwards, a *p*- and an *n*- composition, namely samples with nominal *x* = 0.80 and 0.50, were densified in order to obtain suitable specimens to be submitted to the evaluation of the thermoelectric performance. To this purpose, both specimens were first ball milled at a rotation speed of 150 rpm for 1 h employing a steel jar and steel balls. Subsequently, they were submitted to spark plasma sintering (SPS) by applying in vacuum (P = 5 × 10^−2^ atm) a pressure of 50 MPa at 773 K for 20 min. The discs having 10 mm diameter and 2 mm thickness were obtained. The density of the obtained samples was calculated from their mass and dimension.

The samples were named Fe50_bulk, Fe50_SPS, and so on, according to the % Fe amount with respect to the total (Fe + Ni) content, and to the treatment they were submitted to (annealing: _bulk; annealing followed by SPS: _SPS).

### 2.2. Optical and Electronic Microscopy

The morphology, microstructure, porosity and composition were evaluated by analyzing micrographically polished surfaces of all the samples through optical and electron microscopy. In particular, the specimens were observed by an optical microscope (OM, Leica MEF4M, Leica, Wetzlar, Germany) equipped with a computerized image capture and processing software (Zeiss Axiovision 4, Zeiss, Oberkochen, Germany). A Zeiss SUPRA 40 VP-30-51 scanning electron microscope (Zeiss, Oberkochen, Germany) with a field emission gun (FE-SEM), equipped with a high sensitivity ‘InLens’ secondary electron detector and with an EDS microanalysis INCA Suite Version 4.09 (Oxford Instruments, Abingdon-on-Thames, UK), was employed to study the morphology and evaluate the composition of each phase. The EDS data were optimized using Co as a standard. The microphotographs were taken on each sample both by backscattered and secondary electrons, and the EDS analyses were performed on at least five points (acquisition time: 60 s; working distance: 8.5 mm). The porosity of the bulk and SPS samples was evaluated by image analysis applied both to optical and electron microscopy microphotographs using the Fiji-ImageJ, version 1.49b [56,57].

### 2.3. X-ray Diffraction

All the bulk and SPS samples were powdered, sieved through a 44 μm sieve, placed on a Si zero-background sample holder, and subsequently analyzed by a Bragg-Brentano powder diffractometer (Philips PW1050/81, Fe-filtered Co K_α_ radiation) (Philips, Amsterdam, Netherlands). The diffraction patterns were collected in the angular range 15°–120°, with angular step 0.02°, and counting time 17 s. The structural model of all the occurring phases was refined by the Rietveld method using the FullProf software [58]. The accurate lattice parameters of the skutterudite were obtained from diffractograms collected making use of Ge as an internal standard.

### 2.4. Microhardness

The microhardness of the samples Fe50_bulk, Fe50_SPS, Fe80_bulk and Fe80_SPS was evaluated by a Leica VMHT30A microhardness tester (Leica, Wetzlar, Germany) provided with Vickers indenter. The indentations were done on polished and unetched samples at random positions. Further, 20 tests were performed on each sample applying a test load of 50 g for 15 s. The errors associated with the microhardness average values corresponded to the standard deviation.

### 2.5. Measurements of Transport Properties

The thermal diffusivity (α) and specific heat ($C_{p}$) were measured by a laser flash method using a Netzsch Hyperflash 467 under flowing N_2_ gas (Netzsch, Selb, Germany). A pyroceram standard was used as the specific heat reference. Prior to the measurements, the samples and standard were evenly coated with a thin layer of graphite. For each temperature, 5 data points were obtained and averaged. The SPS samples, with diameter of ~10 mm and thickness of ~2 mm were used directly, while bulk samples were cut into ~6 × 6 × 2 mm^3^ square plates for the measurement. Thermal conductivity (λ) was calculated from the measured thermal diffusivity, specific heat, and density (d) using the relationship:

(3) $$\lambda =\alpha C_{p}d$$

The cuboids with dimensions of ~2 × 2 × 8 mm^3^ were cut from both SPS and bulk samples and used for the Seebeck and electrical resistivity measurements. The simultaneous measurements of Seebeck and the electrical resistivity were performed on a ZEM-2 apparatus (ULVAC-Riko, Chigasaki, Japan) under a low pressure of He atmosphere. The average relative uncertainties of the measurements were 6%, 8%, 11% and 19% for the Seebeck coefficient, electrical resistivity, thermal conductivity, and ZT, respectively [59].

## 3. Results and Discussion

### 3.1. Compositional and Morphological Characterization

According to SEM microphotographs, the EDS analyses and X-ray diffraction, the filled skutterudite results were by far the main phase for each formulation, even if small amounts of extra phases could be revealed in all the samples.

**Figure 1:**
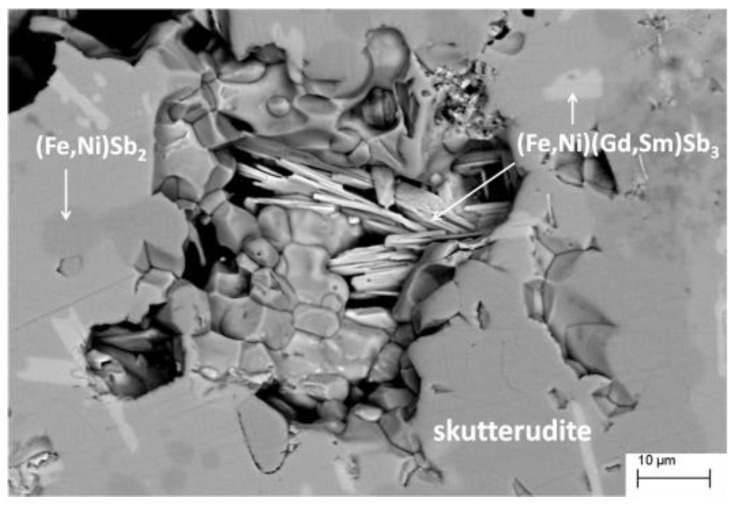
SEM microphotograph taken by backscattered electrons on the polished surface of sample Fe90_bulk.

In Table 1, the experimental composition of each specimen, as obtained from Rietveld refinements, is reported. Due to the similarity in the X-ray scattering factor of Fe and Ni, the Fe/Ni ratio of skutterudite was not refined, but fixed to the value provided by the EDS measurements. The Sm and Gd amounts derive from the refinement of the occupancy factor of the 2a site, and they are in good agreement with the EDS results. It can be observed that in all the samples, the experimental Fe and Ni content were very close to the nominal values. The third column of the Table collects a list of the additional phases identified for each composition. An estimate of the weight percent is reported only when the extra phase could be refined and their amount evaluated by the Rietveld method. When, on the contrary, the presence of the phase could only be revealed by the EDS, but not by X-ray diffraction, its amount is defined as traces. It can be noticed that the most commonly occurring additional phases were Sb and the binary compound (Fe,Ni)Sb_2_, and to a minor extent (Gd,Sm)Sb_3_ and the ternary compound (Ni,Fe)(Gd,Sm)Sb_3_. Among the agreement factors of the Rietveld refinements, R_B_ of skutterudite was quite low, so confirming the accuracy of the structural model. The relatively high values of *χ*^2^ for samples Fe70_bulk and Fe50_bulk are attributable to the very low amount of the secondary phase (Fe,Ni)Sb_2_, which does not allow to correctly refine the model, in particular the Fe/Ni ratio. Regarding Fe80_SPS, the significant decrease of Sb with respect to Fe80_bulk, accompanied by the increase in (Fe,Ni)Sb_2_ and occurrence of traces of (Sm,Gd)_2_Sb_5_ (see Table 1), could result from the effect of vacuum environment during SPS process. The considerably high vapor pressure of Sb at the sintering temperature, coupled to the vacuum condition, could have promoted the reaction of the free Sb present in Fe80_bulk with Fe and Ni deriving from skutterudite. The albeit limited presence of (Gd,Sm)-containing extra phases allows the conclusion that the rare earth content of the filled skutterudite is an intrinsic property of each composition, which is independent of the availability of the filler ions. Figure 1 shows, as a representative example, a microphotograph taken by backscattered electrons on the sample Fe90_bulk, where the presence of the skutterudite-based matrix and small amounts of extra phases can be clearly identified. The figure, moreover, allows the recognition that the average skutterudite crystal size ranges between 5 and 10 μm.

### 3.2. Structural Features

**Figure 2:**
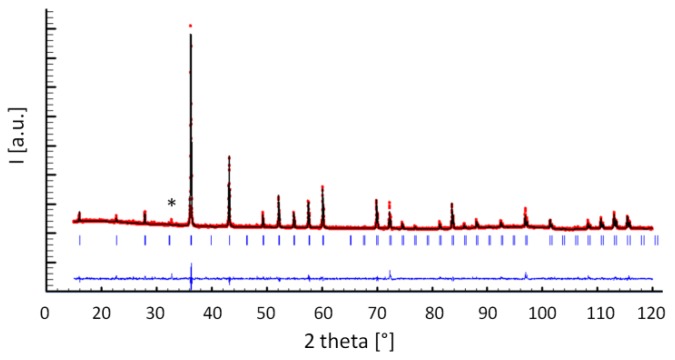
The Rietveld refinement plot of the sample Fe63_bulk. The red and black lines are the experimental and the calculated diffractogram, respectively; the lower line is the difference curve; the blue vertical bars indicate the calculated positions of Bragg peaks of the filled skutterudite; the asterisk (*) indicates the position of the main peak of Sb.

Each X-ray powder diffraction pattern was treated by refining the structural model of the skutterudite. To this purpose, the previously described cubic cell crystallizing in the $Im\bar{3}$ space group was considered. When possible, even the structural models of the additional phases Sb and (Fe,Ni)Sb_2_ were optimized. For every diffractogram, ~70 experimental points chosen from the collected pattern were fitted in order to model the background, while the peak profiles were optimized by means of the pseudo-Voigt function. In the last refinement cycles, the structural parameters of the skutterudite (i.e. the cell parameter, the *x* and *y* atomic coordinates of Sm, and the Sm and Sb occupation in the 2*a* and 24*g* positions, respectively), as well as its scale factor, nine peak parameters, and the background points, were refined at the same time. The individual isotropic displacement parameters B_iso_ were optimized for each atom while keeping the fixed atomic occupancy factors. As aforementioned, Fe and Ni occupancy factors were fixed to the value deriving from the EDS analysis and were not allowed to vary. Figure 2 represents the Rietveld refinement plot of sample Fe63_bulk. The agreement factors are collected in Table 1.

**Figure 3:**
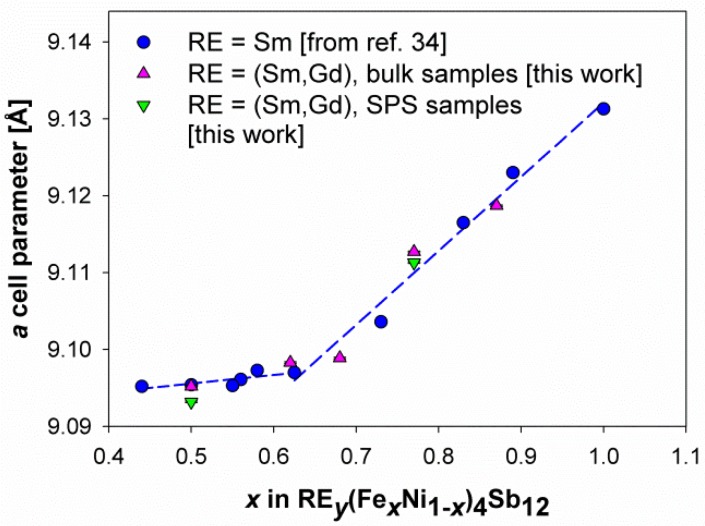
The trend of the lattice parameter of the bulk and SPS samples belonging to the (Sm,Gd)_*y*_(Fe_*x*_Ni_1*-x*_)_4_Sb_12_ as a function of the Fe amount, compared to the values obtained from the Sm_*y*_(Fe_*x*_Ni_1*-x*_)_4_Sb_12_ system (taken from [34]). The dashed lines are regression lines fitting experimental data deriving from [34]. The error bars are hidden by data markers.

The trend of the lattice parameter as a function of the Fe content (*x* in (Sm,Gd)_*y*_(Fe_*x*_Ni_1*-x*_)_4_Sb_12_) is reported in Figure 3. The presence in the same graph of the experimental values obtained from Sm_*y*_(Fe_*x*_Ni_1*-x*_)_4_Sb_12_ [34] allows for an immediate comparison between the two systems and a direct analysis of the effect of Gd addition. Firstly, it can be observed that the lattice parameter grows with increasing the Fe value, owing to the larger size of the latter with respect to Ni, and also to the increasing amount of the filler ion hosted within the Sb_12_ cage with increasing *x*. Moreover, similarly to what revealed in the Sm-containing skutterudite, even if in the presence of a limited number of compositions, the trend of the experimental data suggests the existence of a slope change occurring close to the *p*/*n* crossover, possibly attributable to the Fe^2+^ high- to low-spin transition occurring with increasing *x*, as widely discussed in [34]. On the whole, it can be concluded that no significant differences in the lattice parameter can be appreciated between the samples belonging to both series. Nevertheless, the non negligible difference in the ionic size of Sm^3+^ and Gd^3+^ (r_Sm3+_ [CN12] = 1.24 Å; r_Gd3+_ [CN9] = 1.107 Å) [60] allows the hypothesis that the Gd filler ion could be less strongly retained within the Sb_12_ cage than Sm, with what is in principle expected to be responsible for a lower filling degree of the void in (Sm,Gd) double-filled samples.

**Figure 4:**
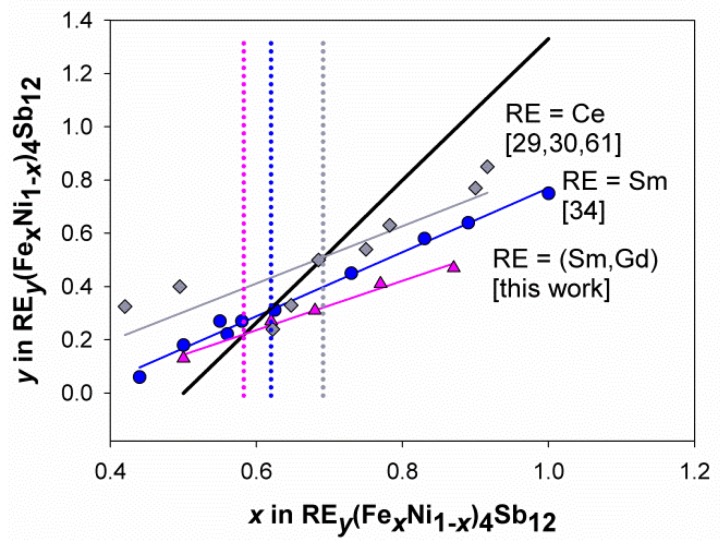
The rare earth content as a function of the Fe amount in different (Fe-Ni)-based skutterudites. The black thick line represents the theoretical amount of trivalent rare earth necessary to reproduce the electronic count of the compensated semiconductor CoSb_3_; experimental values (deriving from this work and from references [29,30,34,61] are fitted by regression lines. The dotted vertical lines pinpoint the position of the *p*/*n* crossover for the different systems.

With this in mind, the diagram appearing in Figure 4 has been built, showing the refined values of the total lanthanide content as a function of the Fe content [*y* vs. *x* in (Sm,Gd)_*y*_(Fe_*x*_Ni_1*-x*_)_4_Sb_12_]. The refined values of the 2*a* site occupancy factor obtained from the only Sm- [34] and Ce-containing system [29,30,61] are reported too as terms for comparison. It has to be underlined that the cited representation is of primary importance for the investigation of the skutterudite properties, since it is able to act as a bridge between the structural and electronic features of the material. As aforementioned, in fact, the filler ion provides a certain number of electrons—according to its oxidation state and amount—which determines the filling fraction of the cavity, expressed in Figure 4 as the occupancy factor of the 2*a* site. The black line also appearing in the figure represents for each x value the filling fraction of the cavity which would ensure the electronic count of a compensated semiconductor. The crossing point between the regression line fitting experimental data and the aforementioned black line, represents the *p*/*n* crossover: At *x* higher than the one corresponding to the crossover, the experimental points lie below the black line, meaning that—at least relying on structural data—a *p*-type conduction regime occurs. The opposite happens at *x* lower than the crossing point. The measurements of the room temperature Seebeck coefficient nicely confirm the accuracy of this determination for Sm_*y*_(Fe_*x*_Ni_1*-x*_)_4_Sb_12_ [34] and Ce_*y*_(Fe_*x*_Ni_1*-x*_)_4_Sb_12_ [29].

Having said that, the analysis of Figure 4 indicates that the (Sm,Gd)-based system presents for each x a rare earth amount significantly lower than the Sm- and the Ce-containing one, leading to roughly parallel regression lines for the three series. The reciprocal position of the regression line with respect to the one of the compensated semiconductor suggests that in the doubly filled system, the *p*/*n* crossover takes place at the lowest Fe amount, namely at *x*~0.59, being *x*~0.63 and *x*~0.70 the crossover points for Sm_*y*_(Fe_*x*_Ni_1*-x*_)_4_Sb_12_ and Ce_*y*_(Fe_*x*_Ni_1*-x*_)_4_Sb_12_, respectively. These informations are indeed of practical relevance, since they have to be taken into account when designing the thermoelectric device. However, they are even more interesting when considering the viewpoint of basic science, in particular when the question to be answered is: Which contributions drive the filling degree of a filled skutterudite?

**Figure 5:**
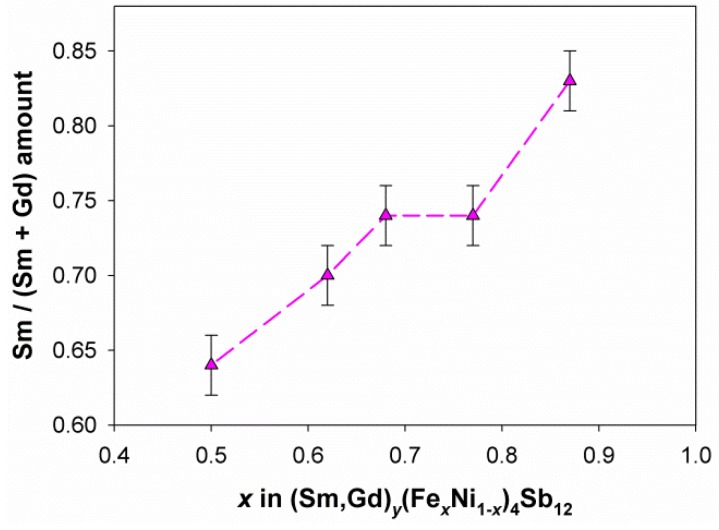
The trend of the refined Sm/(Sm + Gd) amount ratio as a function of the Fe content.

As aforementioned in the Introduction, it is difficult to force into the structure more filler ions than needed to provide the electronic stability to the resulting skutterudite. Therefore, in different RE_*y*_(Fe_*x*_Ni_1*-x*_)_4_Sb_12_ systems, a rough similarity among the content of fillers in the same oxidation state can be observed at a given *x* value, particularly in the compositional region close to the *p*/*n* crossover [28,29,30,31,32,33,34]. On the contrary, significantly different values are observed when alkaline earth ions are inserted, since the 2^+^ oxidation state results into a higher filler amount allowed into the structure [28]. Nonetheless, even if the main driving force ruling the filling fraction is the filler oxidation state, the size effect cannot be neglected. The data reported in Figure 4 clearly show that the filling fraction decreases at each x value following the sequence Ce→Sm→(Sm,Gd), i.e. with decreasing the filler ionic size. According to Shannon [60], in fact, ionic radii are 1.34 Å (CN12), 1.24 Å (CN12) and 1.107 Å (CN9) for Ce^3+^, Sm^3+^ and Gd^3+^, respectively. The described evidence thus suggests that the larger the ions, the stronger their interaction with the cavity, and consequently the higher their content. Moreover, this effect forces the *p*/*n* crossover to shift toward higher *x* values with increasing the ionic size of the filler. In view of the above, the low filler content observed at each *x* value in the (Sm,Gd)-based system can be attributed to the small size of Gd, which makes the (Sm,Gd) mixture on the whole the least retained within the void among the systems considered. This hypothesis can be also discussed in the light of the trend of the Sm/(Sm + Gd) amount ratio as a function of *x*, reported in Figure 5. The almost regular increase in the aforementioned amount ratio with increasing the Fe content, and hence the cavity size [34], is due to the progressive growth of the Sm content, accompanied by the substantially constant amount of Gd, as can be inferred from the refined compositions reported in Table 1. In particular, considering that during synthesis Sm and Gd were added in the 2:1 ratio, the analysis of Figure 5 suggests that the experimental rare earths ratio starts deviating from the nominal value already for *x* = 0.63, where it results equal to 0.7, and it reaches 0.83 for *x* = 0.90. The contribution of the filler ion size to the filling fraction and to the position of the *p*/*n* crossover is therefore further confirmed.

Relying on the present results, it can be thus concluded that multi-filling involving the addition of a small rare earth filler (i.e., a filler which significantly lowers the filling fraction) is expected to exert a two-fold effect. On one hand, it leads to a more effective phonon scattering due to its large atomic mass and small ionic radius, which bring about low frequency modes well interacting with phonons responsible for heat flow in solids. On the other hand, such small ions are weakly bound to the Sb_12_ cage, so that their filling degree is lower than expected for larger, isovalent ions. The latter factor is in principle detrimental, as a large filling degree is desirable, being a regular decrease in the phonon contribution to thermal conductivity generally observed with increasing the filling fraction [62]. Therefore, the choice of filler ions and their proportion is a crucial point in designing multi-filled skutterudites, and it needs to be carefully operated, in order to finely tune the contribution to phonon scattering of fillers with different sizes, keeping in mind the final goal of minimizing thermal conductivity.

### 3.3. Densification of Samples

**Figure 6:**
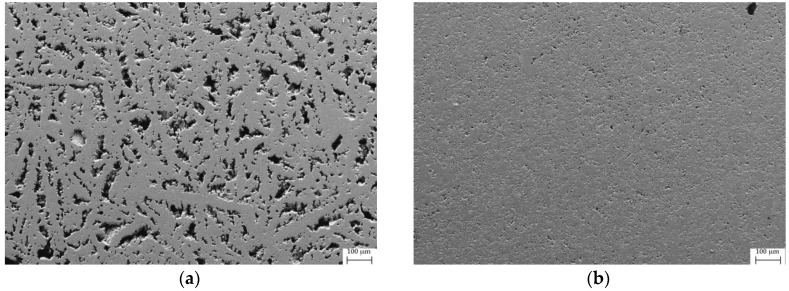
SEM microphotograph taken by secondary electrons on the polished surface of sample (**a**) Fe80_bulk and (**b**) Fe80_SPS.

**Figure 7:**
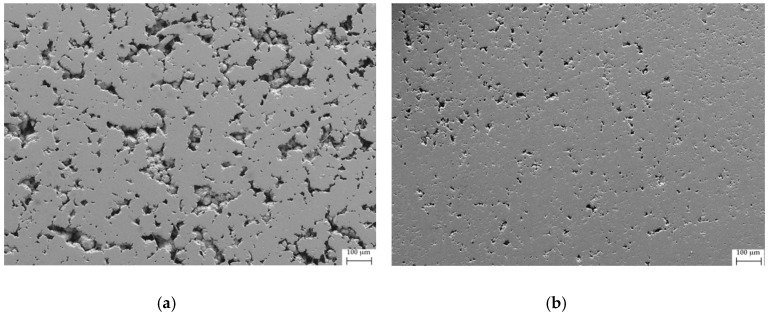
SEM microphotograph taken by secondary electrons on the polished surface of sample (**a**) Fe50_bulk and (**b**) Fe50_SPS.

The effect of SPS on the samples Fe80 and Fe50 was evaluated by considering porosity, microhardness and density of the samples before and after the treatment. In Figure 6 and Figure 7, the microphotographs taken by the secondary electrons prior to and after SPS on the polished surface of the samples Fe80 and Fe50, respectively, are shown. A substantial reduction of porosity can be clearly observed, as also confirmed by the results of the image analysis reported in Table 2, together with a significant density increase. Even the results of the Vickers microhardness measurements point at a hardness increase due to the SPS treatment, as a consequence of the porosity reduction, which limits the probability of including holes within indentations.

The application of an external pressure is possibly expected to reflect also on the structural, microstructural and compositional features of a material. Focusing on skutterudites, different densification methods resulted to affect the lattice parameter [42,63], filling fraction [64], peak width [42], and the amount of additional phases [51]. The diffraction data collected on both as-sintered Fe80 and Fe50 samples, indicate that, in addition to the non-negligible variation in the amount of extra phases in sample Fe80 upon densification (see Table 1), the main change caused by SPS consists of a significant reduction of the lattice parameter as a consequence of the treatment ($\frac{\Delta a}{a}=$ − 1.5 × 10^−4^ for Fe80 and $\frac{\Delta a}{a}=$ − 2.2 × 10^−4^ for Fe50, where a is the lattice parameter), as can be derived from Figure 3. This result is not obvious. While in fact the diffraction patterns collected under the pressure account for a reduction of the cell volume which depends on the bulk modulus of the material [65], the same does not necessarily apply when the acquisition takes place after the pressure relief. An increase in the lattice parameter versus the applied pressure was for instance reported for (Ba,DD,Yb)_*y*_(Fe_1*-x*_Ni_*x*_)_4_Sb_12_ [42], Pr_*y*_(Fe_1*-x*_Ni/Co_*x*_)_4_Sb_12_ and Ba_*y*_Co_4_Sb_12_ [33]. Considering that, according to the results of the Rietveld refinements, the filling fraction does not change upon densification, neither does the global composition of the skutterudite (see Table 1), and the observed decrease in the lattice parameter can be ascribed to a memory effect of the material. However, the analysis of the peak width before and after SPS, showing no significant peak broadening, allows the exclusion of any sign of the grain size reduction, at least as far as the technique can reveal. Therefore, relying on the described results, the densification results were mainly responsible for a porosity reduction and for an enhancement of grain connection, which should favor electrical conductivity. No reduction of thermal conductivity due to scattering of phonons on the defects is expected to occur as a consequence of densification. On the contrary, an increase in the lattice thermal conductivity cannot be excluded as a consequence of the reduction in the amount of pores, which constitute an alternative way of phonon scattering.

### 3.4. Thermoelectric Properties

**Figure 8:**
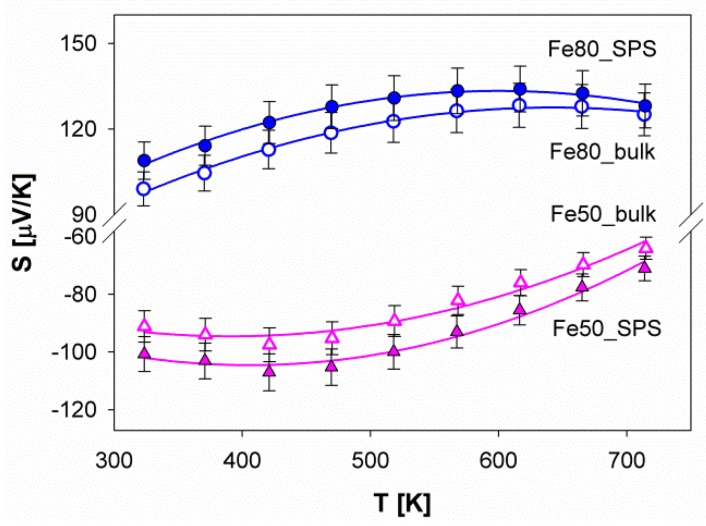
The trend of the Seebeck coefficient as a function of temperature for samples Fe80 and Fe50. The curves fitting the experimental points are second order polynomial functions.

The behavior of the Seebeck coefficient as a function of temperature is reported in Figure 8 for both samples. It can be immediately recognized that the sign of S confirms the *p*- and *n*- nature of Fe80 and Fe50, respectively. Moreover, |S| of the SPS and of the bulk sample are quite close to each other for both compositions as generally expected from samples only differing by their microstructure. The slight enhancement of |S| for SPS with respect to the bulk samples has most probably been attributed to the change in the type and the amount of secondary phases occurring during densification.

The role of the filler on S can be discussed considering that data obtained in this work are essentially comparable to the ones derived from similar systems, such as Sm_*y*_(Fe_*x*_Ni_1*-x*_)_4_Sb_12_ [50] and (Ba,Sr,DD,Yb)_*y*_(Fe_1*-x*_Ni_*x*_)_4_Sb_12_ [42]. Moreover, the room temperature data are in good agreement with the results derived from (Ce,Yb)_*y*_(Fe_*x*_Ni_1*-x*_)_4_Sb_12_, (Ce,Yb)_*y*_(Fe_*x*_Co_1*-x*_)_4_Sb_12_, Ce_*y*_(Fe_*x*_Co_1*-x*_)_4_Sb_12_, Ce_*y*_(Fe_*x*_Ni_1*-x*_)_4_Sb_12_, Yb_*y*_(Fe_*x*_Co_1*-x*_)_4_Sb_12_ and Yb_*y*_(Fe_*x*_Ni_1*-x*_)_4_Sb_12_ systems described in [30], thus suggesting that the Seebeck coefficient is ruled by the charge carriers concentration, rather than by the identity of the filler.

A general feature of semiconductors consists in the occurrence of two different regions in the trend of the absolute value of the Seebeck coefficient versus the temperature, namely an increasing and a decreasing trend at low and high temperatures, respectively, separated by a maximum [42]. This evidence, which is generally accompanied by a similar behavior in the electrical resistivity, can be revealed if the measurement of S extends over a sufficiently broad temperature range, as is the case for our samples. The identification of the maximum value of S ($S_{max}$), as well as of the corresponding temperature ($T_{max}$), is of primary importance, since it allows an estimation of the value of the energy gap ($E_{g}$), according to the following Equation, developed by Goldsmid and Sharp [66]:

(4) $$S_{max}=\frac{E_{g}}{2eT_{max}}$$

being e the electron charge. The data of $T_{max}$, $S_{max}$ and $E_{g}$ for each sample are collected in Table 3; $T_{max}$ and $S_{max}$ were calculated by fitting experimental data to a second order polynomial function.

**Figure 9:**
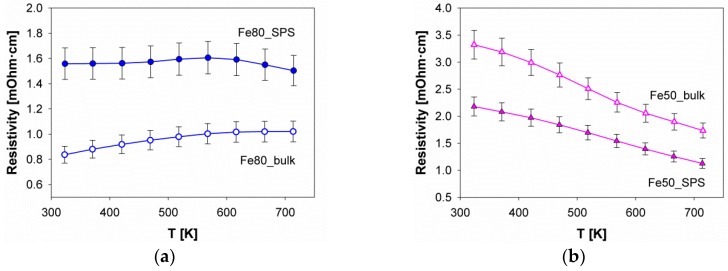
The trend of the electrical resistivity as a function of temperature for sample (**a**) Fe80 and (**b**) Fe50. The curves fitting experimental points are splines.

The trend of the electrical resistivity of samples Fe80 and Fe50 is reported in Figure 9. As a general remark, it can be observed that both compositions exhibited a weak dependence on temperature. Further, despite the weak temperature dependence and the comparatively quite large error bar, a difference can be at a first glance noticed between the two analyzed samples. However, Fe50 shows a semiconducting behavior over the whole temperature range (323 K–723 K), and in Fe80, a plateau-like maximum occurs at a temperature slightly lower than 600 K, meaning that the sample behaves like a bad metal below this temperature, and like a semiconductor above. This is a phenomenon observed at comparable temperatures even in other Fe/Ni-based p-type skutterudites [36,42], which can be explained considering that at lower temperatures the conduction electrons are scattered into unoccupied states. When the conduction band becomes fully occupied, the temperature has to be further increased in order to promote charge carriers across the energy gap, which results in the observed semiconducting behavior at higher temperatures. However, a closer inspection of Figure 9b reveals that in Fe50, the resistivity trend versus the temperature presents two different decreasing rates, being the slope steeper at T< ~400 K than above. This evidence suggests an analogy with the behavior of other *n*-type (Fe/Ni)-based skutterudites, such as (Ba,Sr,DD,Yb)_*y*_(Fe_1*-x*_Ni_*x*_)_4_Sb_12_ (x = 2.1 and 2.2) [42] and DD_0.08_Fe_2_Ni_2_Sb_12_ [38], which exhibit a similar slope change between 450 K and 550 K, and a maximum in the 300 K–400 K range. A change in the conduction regime can be thus inferred even for these compositions, not differently from what happens for the corresponding p-type compounds. The temperature at which the maximum in resistivity occurs derives from a subtle interplay among different factors dealing with the density of states at the Fermi level at each temperature, and with the width of the energy gap. The latter contribution can be estimated exploiting the aforementioned dependence of the energy gap ($E_{g}$) on the maximum value of the Seebeck coefficient versus the temperature ($S_{max}$), i.e., through Equation (4). The calculated values of $E_{g}$ are 160 meV and 88 meV for samples Fe80_SPS and Fe50_SPS, respectively, as reported in Table 3. The rather smaller value of the calculated $E_{g}$ for the n-type compound justifies the possible existence of a maximum in the resistivity behavior at a significantly lower temperature than the *p*-type one.

Further, regarding electrical resistivity, it is also worth mentioning that, while in sample Fe50 a higher value occurs for the bulk sample than for the SPS one, the opposite happens for Fe80. This evidence, also noticed for the Sm_*y*_(Fe_1*-x*_Ni_*x*_)_4_Sb_12_ system [51], is most probably due to the competition between two opposing effects taking place as a consequence of densification, namely an improvement of grain connection, and an increase in the defects amount. While the former promotes a resistivity decrease, the opposite happens for the latter. The behavior observed for Fe80, namely lower resistivity values for the bulk sample, can be ascribed to a couple of different factors. First of all, a possible reason is the relatively scarce density degree reached (88%, significantly lower than 97% obtained for Fe50_SPS, see Table 2), which may favor the predominance of the defect creation over the improvement of the grain connection. Nonethless, a positive effect of porosity on ZT of Co_1*-x*_Ni_*x*_Sb_3_ was already observed by He et al. [67]. Secondarily, since the Fe80 sample contains a non negligible amount of a secondary phase, the electrical resistivity of this sample could be also influenced by the properties of the dispersed secondary phase. Prior to SPS, the sample Fe80_bulk contains a certain amount of antimony (11 wt %), which possesses a relatively low value of electrical resistivity (ρ_295.5K_ = 34.9 µΩ cm [68]). SPS caused the removal of the majority of antimony, and (Fe,Ni)Sb_2_, namely a semiconductor with a higher value of electrical resistivity compared to Sb [69] emerged as the dominant secondary phase. The reduced amount of metallic Sb together with the increased amount of semiconducting (Fe,Ni)Sb_2_ is a further possible reason why the electrical resistivity is higher in Fe80_SPS than in Fe80_bulk.

**Figure 10:**
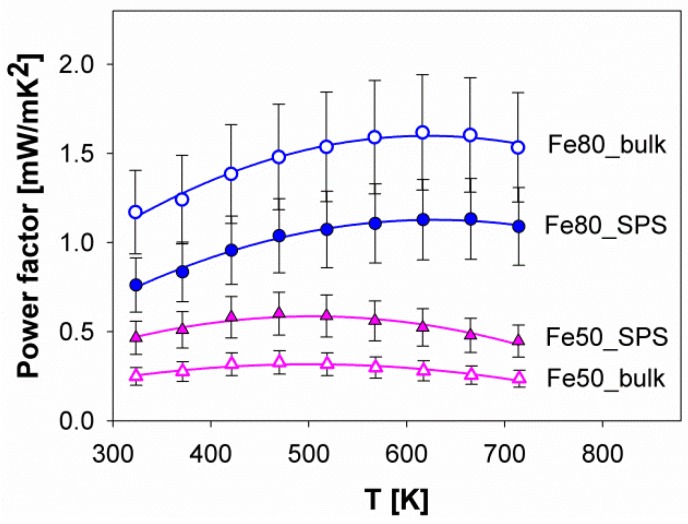
The trend of the power factor as a function of the temperature for samples Fe80 and Fe50. The curves fitting the experimental points are second order polynomial functions.

Combining electrical conductivity and the Seebeck coefficient allows the calculation of the power factor (PF) at each temperature considered. In Figure 10, the PF values of the samples Fe80 and Fe50 are reported. For both compositions, the temperature at which the maximum power factor occurs is slightly higher than the one corresponding to the maximum value of the Seebeck coefficient. The obtained trends of PF versus the temperature can be compared to the data deriving from similar Sb-based filled skutterudites, such as (Ba,Sr,DD,Yb)_*y*_(Fe_1*-x*_Ni_*x*_)_4_Sb_12_ [42]. It can be observed that values reported for the aforementioned system both at *x* = 0.50 and 0.80 are higher than the ones claimed in this work.

This evidence can be most probably be ascribed to the lower values of electrical resistivity of the multi-filled compounds, rather than to the Seebeck coefficient. As previously described, in fact, S values of the systems containing even different fillers are quite close to each other, pointing at a dependence of the Seebeck coefficient mainly on electronic issues. This interpretation is confirmed by the analysis of the data collected at 350 K on (Sm,Gd)_0.15_(Fe_0.5_Ni_0.5_)_4_Sb_12_ (this work) and Yb_*y*_(Fe_0.5_Ni_0.5_)_4_Sb_12_ [43]. At this temperature, the former system is characterized by a higher power factor when compared to each composition of the latter. Again, the electrical resistivity seems to be responsible for this dissimilarity, being significantly lower in (Sm,Gd)_0.15_(Fe_0.5_Ni_0.5_)_4_Sb_12_ than in Yb_*y*_(Fe_0.5_Ni_0.5_)_4_Sb_12_. It can be thus concluded that the improvement of the power factor is mainly related to the improvement of electrical conductivity, which can be accomplished by optimizing the material processing.

**Figure 11:**
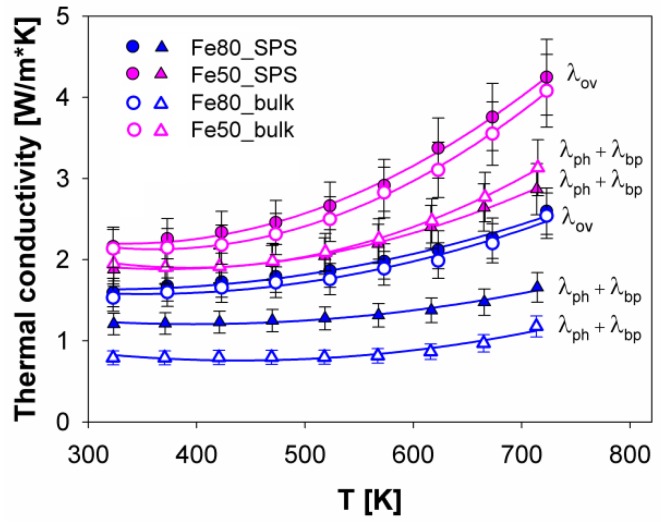
The trend of overall ($\lambda_{ov}$), and lattice and bipolar ($\lambda_{ph}+\lambda_{bp}$ ) thermal conductivity of the samples Fe80_SPS and Fe50_SPS as a function of the temperature.

The trends of overall ($\lambda_{ov}$), and phonon and bipolar ($\lambda_{ph}+\lambda_{bp}$) thermal conductivity of Fe80 and Fe50 are reported in Figure 11. While ($\lambda_{ph}+\lambda_{bp}$) of Fe50 does not significantly change with densification, it increases for Fe80. The latter behavior is generally expected, since pores can offer a further way for phonon scattering.

It can be observed that $\lambda_{ov}$ increases with increasing temperature for both samples, but more steeply for Fe50_SPS, with 1.6 W/m·K and 4.2 W/m·K the minimum and maximum value in the 323 K–723 K temperature range. As already revealed for DD_*y*_(Fe_*x*_Ni_1*-x*_)_4_Sb_12_ [38], even in the (Sm,Gd)_*y*_(Fe_*x*_Ni_1*-x*_)_4_Sb_12_ system, thermal conductivity is lower at *x* = 0.8 than at x = 0.5. This result can be understood considering that the thermal conductivity reduction arises from the two-fold effect of a) the rattling motion of the filler, which is responsible for the *λ* reduction at low temperatures, and b) the disorder brought about by the contemporary presence of two different atoms at the 8c position (Fe and Ni in the present study) [70]. By relying on the data reported in [70], this combined effect causes the minimum lattice thermal conductivity to be located at *x* ~1.5 in Ce_*y*_Fe_4*-x*_Co_*x*_Sb_12_, which corresponds to *x* = 0.80 in (Sm,Gd)_*y*_(Fe_*x*_Ni_1*-x*_)_4_Sb_12_, if Fe is taken as a bivalent and Ni as a tetravalent ion, as suggested in [18].

The effect of the sole filler on thermal conductivity can be studied by comparing antimonides having the same (Fe/Ni) amount ratio. For instance, the comparison between $\lambda_{ov}$ of (Sm,Gd)_*y*_(Fe_*x*_Ni_1*-x*_)_4_Sb_12_ and Sm_*y*_(Fe_*x*_Ni_1*-x*_)_4_Sb_12_ [50] with x = 0.8, points at a lower value for the former system, as a consequence of double filling. An analogous comparative approach applied to $\lambda_{ov}$ of (Sm,Gd)_*y*_(Fe_*x*_Ni_1*-x*_)_4_Sb_12_ with x = 0.5 and Yb_*y*_(Fe_0.5_Ni_0.5_)_4_Sb_12_ [43] reveals similar values in the two systems, at least in the 350 K–650 K temperature range. This evidence, which at a first glance could seem contradictory, finds an explanation when the valence state of Yb is taken into account. According to X-ray absorption spectroscopy measurements performed on (Ce-Yb)_*y*_Fe_4*-x*_Co_*x*_Sb_12_ and (Ce-Yb)_*y*_Fe_4*-x*_Ni_*x*_Sb_12_ [30], Yb occurs in a +2/+3 mixed valence state, evolving toward +3 with decreasing the filling degree. This intermediate valence state leads a Yb-containing skutterudite to mimic a double-filled system, as a consequence of the different size of Yb^2+^ and Yb^3+^, which can justify the observed very similar values of thermal conductivity of the Yb- and the (Sm,Gd)-filled skutterudite.

Assuming the validity of the Wiedemann-Franz law:

(5) $$\lambda_{el}=\frac{L_{0}T}{\rho }$$

where $L_{0}$ is the Lorenz number (calculated using the Seebeck coefficient, according to [71]), it is possible to give an estimation of the sum of lattice and bipolar thermal conductivity ($\lambda_{ph}+\lambda_{bp}$) by subtracting $\lambda_{el}$ from $\lambda_{ov}$. The values reported in Figure 11 were obtained this way. It is noteworthy that ($\lambda_{ph}+\lambda_{bp}$) increases with increasing temperature similarly to $\lambda_{ov}$, but the increasing rate is much weaker, particularly in the high temperature region. This result suggests that with increasing temperature $\lambda_{el}$ becomes the main contribution to thermal conductivity. If $\lambda_{ph}$ of the (Ba,DD,Yb)_*y*_(Fe_1*-x*_Ni_*x*_)_4_Sb_12_ system with x = 0.78 [42] is considered, it can be noticed that the values are very close to the ones of Fe80_bulk, in what can be deemed as a very promising result, since it is reached making use of just two fillers.

A closer inspection of the ($\lambda_{ph}+\lambda_{bp}$) trend versus the temperature allows a deeper insight into the role of the bipolar contribution to thermal conductivity of the present system. The cited factor is determined by the electron-hole pairs, which together with electrons and phonons are responsible for the heat transfer in semiconductors. The electron-hole coupling occurs at sufficiently high temperatures depending on the band gap, so that, as aforementioned, $\lambda_{bp}$ becomes relevant only above a certain threshold, which is strictly bound to the system under examination. A useful way to reveal the signature of the electron-hole coupling is to plot ($\lambda_{ov}-\lambda_{el}$) as a function of T^−1^. At low temperatures, a decreasing linear trend with increasing temperature is generally observed due to Umklapp scattering, and the temperature where data start deviating from linearity can be taken as the threshold temperature above which the bipolar contribution becomes significant. The data deriving from Yb-filled Co_4_Sb_12_ follow this model [72].

**Figure 12:**
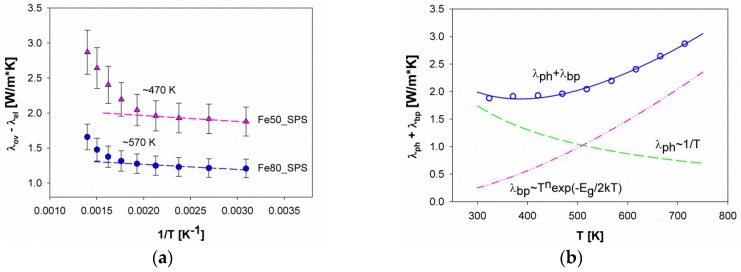
(**a**) The trend of ($\lambda_{ov}-\lambda_{el}$) vs. 1/T for samples Fe80_SPS and Fe50_SPS. The dashed lines are a guide for the eye. The temperatures reported within the diagram roughly indicate the threshold above which the bipolar contribution starts growing steeply; (**b**) fit of ($\lambda_{ph}+\lambda_{bp}$ ) data (continuous line) performed according to Equation 6) (dashed-dotted line) and to Umklapp scattering (dashed line).

Figure 12a reports the described plot for samples Fe80_SPS and Fe50_SPS. It can be seen that ($\lambda_{ph}+\lambda_{bp}$) of both samples tends to increase with increasing temperature, instead of decreasing with the usual T^−1^ temperature dependence, as previously described. Further, above ~570 K for Fe80_SPS and ~470 K for Fe50_SPS, the values start growing more steeply with increasing temperature, in good agreement with the larger $E_{g}$ calculated for the former composition (160 meV) with respect to the latter (88 meV). However, the increasing trend along the whole temperature range suggests that the bipolar contribution near room temperature is already significant and able to mask the contribution of Umklapp scattering. This is particularly relevant for the Fe50_SPS sample, since the extracted thermal band gap is only 88 meV, which is just a few kT at room temperature. In order to quantify the contribution of both processes, a least square fit of the experimental data was performed using the relation for bipolar thermal conductivity

(6) $$\lambda_{bp}=cT^{n}exp\left(-\frac{E_{g}}{2kT}\right)$$

where *c* and *n* are adjustable parameters [4], together with the aforementioned T^−1^ dependency for the high temperature Umklapp scattering. A reasonably good agreement was observed for the Fe50_SPS sample, as shown in Figure 12b. As expected, the contribution of bipolar conductivity amounts to approximately 10% near room temperature and increases significantly at higher temperature. The same approach does not work satisfactorily on Fe80_SPS, presumably due to the presence of secondary phases, that are not accounted for by the described model.

**Figure 13:**
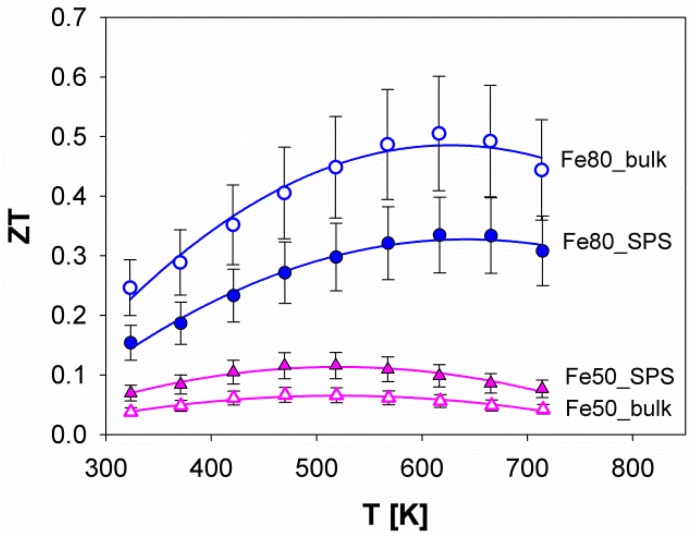
The trend of ZT as a function of the temperature for the samples Fe80 and Fe50. The curves fitting experimental points are second order polynomial functions.

The trend of ZT as a function of the temperature for both Fe80 and Fe50 is shown in Figure 13. First, it can be noticed that Fe80 is characterized by higher ZT values than Fe50 at each temperature, as a consequence of the higher power factor and the lower total thermal conductivity, as previously described. Moreover, the maximum ZT occurs at ~615 K for Fe80, and at ~500 K for Fe50. Interestingly, Fe80_bulk shows higher values of ZT than Fe80_SPS, due to the lower thermal conductivity and to the higher values of the power factor, which are in turn caused by the previously described lower resistivity of the porous sample with respect to the dense one. The comparison between ZT data of Fe80 and the ones of the corresponding Sm-filled sample [50] indicates that the single-filled system presents the maximum at a slightly higher temperature (~615 K), and a lower value of the figure of merit, as a consequence of the higher thermal conductivity. Analogously, a slightly lower value of the figure of merit is shown by Yb_*y*_(Fe_0.5_Ni_0.5_)_4_Sb_12_ at each y value [43] with respect to Fe50, as could be expected from the aforementioned very similar values of thermal conductivity for the two systems. Finally, the higher value of the maximum ZT occurring for the (Ba,DD,Yb)_*y*_(Fe_1*-x*_Ni_*x*_)_4_Sb_12_ system with *x* = 0.78 [42], can be considered as a direct consequence of the previously discussed higher power factor value.

The closeness between the studied system and the described multi-filled (Fe,Ni)-based skutterudites in terms of thermoelectric performance, and in particular of thermal conductivity, allows the conclusion that the selection of the (Sm,Gd) mixture as a filler is a good compromise between the limited Sm^3+^/Gd^3+^ mass difference and the relatively low filling fraction.

## 4. Conclusions

The structural and thermoelectric properties of both *p*- and *n*- compositions belonging to the skutterudite system (Sm,Gd)*_y_*(Fe*_x_*Ni_1-*x*_)_4_Sb_12_ were studied with the aim of evaluating the effect of double filling by Sm and Gd on composition, crystallographic parameters and transport properties of the material.

The results of the structural investigation suggest that the employment of the (Sm,Gd) mixture as a filler causes the filling fraction to be always lower when using Ce or Sm. Moreover, with increasing the Fe amount, the Sm/(Sm + Gd) ratio increases too, suggesting that Sm is preferred when the cage size grows. Both evidences point at a role of the filler size in ruling the filling fraction, in addition to the filler oxidation state.

Despite the Sm^3+^/Gd^3+^ small mass difference, the outcome of the thermoelectric study shows that the contemporary presence of these ions within the void located in the 2*a* site significantly reduces at each temperature considered by the total thermal conductivity of both *p*- and *n*-compositions below the values of the only Sm-filled compound. Moreover, both the phonon thermal conductivity and the overall thermoelectric performance are comparable to those of several multi-filled (Fe,Ni)-based skutterudite antimonides described in the literature. The choice of the (Sm,Gd) mixture as a filler thus results to be a good compromise between the limitations imposed by the reduced Gd^3+^ size and the Sm^3+^/Gd^3+^ mass closeness.

## Figures and Tables

**Table 1:** The refined compositions, list of additional phases, and agreement factors of the Rietveld refinements (χ^2^ and skutterudite R_B_).

Sample	Refined Composition	Additional Phases	χ^2^	R_B_
Fe90_bulk	Sm_0.39(1)_Gd_0.08(1)_(Fe_0.87(1)_Ni_0.13(1)_)_4_Sb_12_	Sb (1 wt.%), (Fe,Ni)Sb_2_ (14 wt.%), (Fe,Ni)(Gd,Sm)Sb_3_ *	6.4	5.8
Fe80_ bulk	Sm_0.31(1)_Gd_0.11(1)_(Fe_0.78(1)_Ni_0.22(1)_)_4_Sb_12_	Sb (11 wt.%), (Fe,Ni)Sb_2_*, (Sm,Gd)_2_Sb_5_ *	4.0	4.0
Fe80_SPS	Sm_0.34(1)_Gd_0.08(1)_(Fe_0.76(1)_Ni_0.24(1)_)_4_Sb_12_	Sb (2 wt.%), (Fe,Ni)Sb_2_ (7 wt.%), (Sm,Gd)_2_Sb_5_ *	4.2	3.0
Fe70_ bulk	Sm_0.23(1)_Gd_0.08(1)_(Fe_0.68(1)_Ni_0.32(1)_)_4_Sb_12_	Sb (11 wt.%), (Fe,Ni)Sb_2_*, Sb_5_(Gd,Sm)_2_ *	14.8	6.9
Fe63_ bulk	Sm_0.19(1)_Gd_0.08(1)_(Fe_0.63(1)_Ni_0.37(1)_)_4_Sb_12_	Sb *, (Fe,Ni)SmSb_3_ *	5.8	4.8
Fe50_ bulk	Sm_0.09(1)_Gd_0.05(1)_(Fe_0.51(1)_Ni_0.49(2)_)_4_Sb_12_	(Fe,Ni)Sb_2_ (1 wt.%), (Ni,Fe)(Gd,Sm)Sb_3_ *	12.2	4.7
Fe50_SPS	Sm_0.08(1)_Gd_0.07(1)_(Fe_0.51(1)_Ni_0.49(2)_)_4_Sb_12_	(Fe,Ni)Sb_2_ (2 wt.%), (Ni,Fe)(Gd,Sm)Sb_3_ *, Ni_0.4_Sb_2_(Sm,Gd) *	6.5	5.8

* traces.

**Table 2:** The porosity, Vickers microhardness, the absolute and relative density of the samples Fe50 and Fe80 before and after the SPS treatment.

Sample	Porosity [%]	Vickers Microhardness	Density [g/cm^3^]	Density [%]
**Fe80_bulk**	19	328(70)	6.40	82
**Fe80_SPS**	10	433(50)	6.87	88
**Fe50_bulk**	16	460(49)	6.62	87
**Fe50_SPS**	4	475(55)	7.33	97

**Table 3:** The maximum value of the Seebeck coefficient ($S_{max}$ ), the temperature corresponding to $S_{max}$ ($T_{max}$ ), and the value of the energy gap ($E_{g}$) estimated through Equation (4) for samples Fe80 and Fe50.

Sample	*S_max_* [μV/K]	*T_max_* [K]	*E_g_* [meV]
**Fe80_bulk**	128	642	164
**Fe80_SPS**	133	600	160
**Fe50_bulk**	−97	409	79
**Fe50_SPS**	−106	413	88
